# Specific Language Impairment in African American English and Southern White English: Measures of Tense and Agreement With Dialect-Informed Probes and Strategic Scoring

**DOI:** 10.1044/2019_JSLHR-L-19-0089

**Published:** 2019-09-13

**Authors:** Janna B. Oetting, Jessica R. Berry, Kyomi D. Gregory, Andrew M. Rivière, Janet McDonald

**Affiliations:** a Department of Communication Sciences and Disorders, Louisiana State University, Baton Rouge; b Speech Pathology and Audiology Department, South Carolina State University, Orangeburg; c Communication Sciences and Disorders Program, Pace University, New York, NY; d Sentara Enterprises, Woodbridge, VA; e Department of Psychology, Louisiana State University, Baton Rouge

## Abstract

**Purpose:**

In African American English and Southern White English, we examined whether children with specific language impairment (SLI) overtly mark tense and agreement structures at lower percentages than typically developing (TD) controls, while also examining the effects of dialect, structure, and scoring approach.

**Method:**

One hundred six kindergartners completed 4 dialect-informed probes targeting 8 tense and agreement structures. The 3 scoring approaches varied in the treatment of nonmainstream English forms and responses coded as Other (i.e., those not obligating the target structure). The unmodified approach counted as correct only mainstream overt forms out of all responses, the modified approach counted as correct all mainstream and nonmainstream overt forms and zero forms out of all responses, and the strategic approach counted as correct all mainstream and nonmainstream overt forms out of all responses except those coded as Other.

**Results:**

With the probes combined and separated, the unmodified and strategic scoring approaches showed lower percentages of overt marking by the SLI groups than by the TD groups; this was not always the case for the modified scoring approach. With strategic scoring and dialect-specific cut scores, classification accuracy (SLI vs. TD) was highest for the 8 individual structures considered together, the past tense probe, and the past tense probe irregular items. Dialect and structure effects and dialect differences in classification accuracy also existed.

**Conclusions:**

African American English– and Southern White English–speaking kindergartners with SLI overtly mark tense and agreement at lower percentages than same dialect–speaking TD controls. Strategic scoring of dialect-informed probes targeting tense and agreement should be pursued in research and clinical practice.

In the current work, we examined the profile of specific language impairment (SLI) within two nonmainstream dialects of English—African American English (AAE) and Southern White English (SWE)—by creating four dialect-informed probes to examine eight different tense and agreement structures and three scoring approaches. In mainstream dialects of English, such as General American English (GAE), tense and agreement structures are difficult for children with SLI and other developmental language disorders (Rice, 2009; Rice, Tomblin, Hoffman, Richman, & Marquis, 2004; Rice & Wexler, 1996; Rice, Wexler, & Hershberger, 1998). As such, these structures are routinely targeted in tests of language, such as the Clinical Evaluation of Language Fundamentals Preschool–Second Edition (Semel, Wigg, & Secord, 2004), Clinical Evaluation of Language Fundamentals–Fifth Edition (CELF-5; Wiig, Semel, & Secord, 2013), Preschool Language Scales–Fifth Edition (Zimmerman, Steiner, & Pond, 2011), and Structured Photographic Expressive Language Test–Third Edition (Dawson, Stout, & Eyer, 2003).

In nonmainstream dialects of English, such as AAE and SWE, expressions of tense and agreement vary, with SWE allowing a greater range of forms than GAE and AAE allowing an even greater range than both GAE and SWE. For example, GAE marks past tense with mainstream overt forms (e.g., *walked*, *drank*). SWE marks past tense with these forms too but allows a low rate of nonmainstream zero forms (e.g., *walkØ*, *drinkØ, seeØ*) and nonmainstream overt forms (e.g., *drinked, drunk, seen*). AAE marks past tense with the same forms as GAE and SWE but allows more zero marking and a larger inventory of nonmainstream overt forms (e.g., *drinked, drunk, seen, fount, had play, had played*). To accommodate dialects that differ from GAE, many tests of language, including those previously listed, encourage clinicians to modify the scoring of nonmainstream forms when they are consistent with a child's dialect.

Modified scoring approaches accommodate children's dialect differences by scoring nonmainstream responses as correct. The practice is endorsed by the American Speech-Language-Hearing Association (2003) because it helps close the gap between scores earned by mainstream and nonmainstream English speakers (Beyer & Hudson-Kam, 2011; Charity, Scarborough, & Griffin, 2004; Terry, Jackson, Evangelou, & Smith, 2010). However, scoring all nonmainstream responses as correct reduces the number of items and potentially entire categories of content (e.g., tense and agreement) that can be used to identify children with SLI. Studies also have not always found modified scoring systems to improve diagnostic accuracy (Craig, Thompson, Washington, & Potter, 2004; Hendricks & Adlof, 2017; Rhyner, Kelly, Brantley, & Krueger, 1999). For example, using data from 77 AAE-speaking second graders, Hendricks and Adlof (2017) compared child outcomes on three subtests of the CELF-5 when items were scored with and without modification. Although modified scoring led to higher scores than unmodified scoring, the diagnostic accuracy of the CELF-5 with either scoring approach was less than ideal. Moreover, whereas the unmodified approach led to high rates of false positives (i.e., overidentification), the modified approach led to high rates of false negatives (i.e., underidentification). Neither outcome is acceptable for clinical practice.

## Tense and Agreement Deficits of AAE- and SWE-Speaking Children With SLI

In pursuit of better practice, Oetting and colleagues have been studying children's development of AAE and SWE as spoken in the rural south while also examining potential differences between children with and without SLI within these dialects. In mainstream dialects of English and different languages, grammar deficits are a feature of the SLI profile (Leonard, 2014), so it reasonable to look for similar SLI deficits in AAE and SWE.

In an early study using language sample data, Oetting and McDonald (2001) examined 62 AAE- and SWE-speaking kindergartners' relative frequencies of 35 different nonmainstream grammar forms (e.g., zero past tense, zero verbal –*s*). Half of the samples were from children with SLI, and the others were from age-matched typically developing (TD) controls.^1^ With only the relative frequencies of the nonmainstream grammar forms, 97% of the kindergartners were accurately classified as either an AAE or SWE speaker, and within each dialect, ≥ 90% were accurately classified as either SLI or TD. Using language samples from the 106 kindergartners in the current study, Vaughn and Oetting (2018) replicated this result (dialect classification = 88%; within each dialect, clinical group classification ≥ 90%). In both studies, clinical group differences (SLI > TD) were also observed for some of the individual tense and agreement forms; these forms included zero BE, DO and irregular past, and in the latter study, which used a slightly more elaborate coding system, regular past tense and verbal *–s*.

Other studies have also found tense and agreement differences between children with and without SLI in AAE and SWE while also showing dialect and dialect by structure differences (Oetting, 2019). For example, Oetting and Garrity (2006) examined AAE- and SWE-speaking children's percentages of zero regular past (e.g., *walkØ*), zero regular verbal –*s* (e.g., *walkØ*), zero *is* (e.g., *he Ø walking*), zero *are* (e.g., *they Ø walking*), and *was* for *were* (e.g., *they was walking*). All five tense and agreement forms showed effects for the children's dialects (AAE > SWE), and two forms (i.e., zero past regular and zero *is*) showed effects for the children's clinical group (SLI > TD). The effect size of the clinical group difference, as measured by Cohen's *d*, was .90 for zero past and .97 for zero *is*. Consistent with the adult AAE and SWE literature, the children's patterning of overt marking also varied by the children's dialect and the tense and agreement structure. Specifically, the AAE speakers overtly marked past tense at higher percentages than verbal –*s* (69% vs. 22%) and *is* at higher percentages than *are* (51% vs. 28%). In contrast, the SWE speakers overtly marked all forms at relatively high percentages, except *are*, which was overtly marked at a lower percentage (past tense 89%, verbal –*s* 83%, *is* 90% vs. *are* 72%).

In another study, Cleveland and Oetting (2013) examined AAE- and SWE-speaking children's overt marking of different types of verbal –*s* structures (i.e., regular vs. irregular as in *jumps* vs. *does*; habitual vs. nonhabitual as in *She always drives us to school* vs. *The baby wants the bottle now*; DO with vs. without negation as in *She doesn*'*t* vs. *She does*). Results showed that the children's percentages of overt marking differed by dialect (AAE < SWE) and structure (e.g., irregular > regular, habitual < nonhabitual, with negation < without negation). Also, in SWE but not AAE, the SLI group produced lower percentages of overt marking than the TD group for all verbal –*s* structures, except when DO was produced with negation. When all structures were combined, the effect size of the SWE SLI versus TD difference as measured by Hedges' *g* was 1.91.

Focusing on AAE, Garrity and Oetting (2010) examined children's overt marking of auxiliary *am, is*, and *are* using language samples and probes. Across tasks and structures, the children with SLI produced the lowest percentages of overt marking, and for the language samples, large effects were observed between the SLI and age-matched TD groups for *is* (Hedges' *g* = 1.36) and *are* (Hedges' *g* = 1.21)*.* The children's patterning of marking by structure also mirrored the adult AAE literature, because percentages of overt marking were highest for *am* (96%), lower for *is* (32%), and lowest for *are* (16%). Finally, in another lab, Seymour, Bland-Stewart, and Green (1998) examined the percentages at which AAE-speaking children with and without SLI produced mainstream overt forms for 17 grammatical structures in language samples. Although four structures led to statistical differences between the groups, only regular past tense led to a difference (SLI < TD) with a large effect (Cohen's *d* = 1.54).

Although the above-reviewed studies have revealed clinical group, dialect, and dialect by structure differences, the findings do not translate well to clinical practice. Most of the studies have focused on language samples that are labor intensive. Moreover, samples need to be especially large (i.e., > 200 child utterances) when a dialect allows variable marking and many different types of mainstream and nonmainstream overt forms and zero forms, as is the case for AAE and SWE. Even when samples are large, the number of tense and agreement forms can be extremely low and unequal across structures and groups, which limits statistical power to detect differences. For example, in the 57 AAE and SWE language samples studied by Cleveland and Oetting (2013), there were 484 habitual verbal –*s* contexts but only 274 nonhabitual contexts, and only 20 nonhabitual verbal –*s* contexts were produced by the AAE SLI group. Whereas a clinical difference (SLI < TD) was found in SWE for nonhabitual verbal –*s*, a similar difference was not found in AAE, and the low number of nonhabitual contexts produced by the AAE SLI group may have contributed to this finding.

## Dialect-Informed Tasks and Strategic Scoring

Moving away from language samples, Oetting, McDonald, Seidel, and Hegarty (2016) developed a sentence recall task that targeted tense and agreement structures in every item. Informed by the AAE and SWE literature and the study of SLI within these dialects, a diverse set of morphemes and main verbs were included to elicit high numbers of tense and agreement structures. The complexity of the sentences was also manipulated by varying the number of functional categories (i.e., tense, negation, and complementizer) within the sentences, because across languages, marking of tense and agreement (and other morphological structures) can be compromised in children when the number of functional categories within an utterance exceeds their processing capacities (Hegarty, 2005). Finally, strategic scoring was implemented by counting as correct three types of nonmainstream responses: *is* for *are*, *was* for *were*, and zero verbal –*s*. These responses were scored as dialect-appropriate because children's use of them has led to consistent effects for dialect (AAE vs. SWE), without consistent effects for clinical group (SLI vs. TD), especially within AAE (Cleveland & Oetting, 2013; Oetting & Garrity, 2006). All other nonmainstream responses were scored as errors to capture any frequency-based differences between the SLI and TD groups.

When compared to same dialect–speaking TD controls, the children with SLI produced fewer exact recalls, more ungrammatical recalls when the recalls were not exact, and higher levels of recall error on tense and agreement structures.^2^ Also, the classification accuracy (SLI vs. TD) of the recall task was relatively high for the dialects combined (Se = .91, Sp = .85) and separated (AAE: Se =.89, Sp = .86; SWE: Se = .94, Sp = .83). These rates of classification accuracy were higher than what was found for these same children on two processing-based tasks, that is, a working memory task of size judgment: total links (dialects combined: Se = .77, Sp = .72; AAE: Se = .74, Sp = .66; SWE: Se = .83, Sp = .83; McDonald, Seidel, Hammarlund, & Oetting, 2018) and a phonological short-term memory task of nonword repetition: percent phonemes correct (dialects combined: Se = .53, Sp = .98; AAE: Se = .43, Sp = .97; SWE: Se = .72, Sp = 1.00; McDonald & Oetting, 2019).

Findings from the sentence recall study demonstrate the potential use of dialect-informed tasks and strategic scoring for clinical practice; however, additional studies are needed to examine other types of tasks and scoring approaches. This work is particularly important for measures of tense and agreement, because as noted previously, children's percentages of overt marking vary by their dialect and the structure examined. Also, further complicating the study of tense and agreement within AAE (and possibly SWE) are effects of linguistic context that influence the likelihood of zero marking. Linguistic context effects are well attested in the adult and child dialect literature (e.g., Garrity & Oetting, 2010; Green, 2011; Pruitt & Oetting, 2009; Rickford, 1999; Rickford, Ball, Blake, Jackson, & Martin, 1991; Wyatt, 1991; for dialects of Spanish, see Miller, 2013; for Creole language varieties, see Wilkerson, 2015). Most relevant for the current study, AAE speakers are more likely to zero mark regular past tense when the context requires a consonant cluster (e.g., *jumped*) than when it does not (e.g., *mowed*). AAE speakers are also more likely to zero mark auxiliary BE when the preceding subject involves a personal pronoun (e.g., *he is walking*) than when it involves a specific noun (e.g. *Jared is walking*).

By using probes rather than language samples, one can control the structures and contexts elicited. Therefore, in the current study, we created four dialect-informed probes to target eight different tense and agreement structures (i.e., two structures per probe). The tense and agreement structures included the often-studied morphemes of regular and irregular past tense and auxiliary *is, are, was*, and *were*, and the less studied habitual and nonhabitual verbal –*s*. The habitual nature of verbal –*s* was of interest because this structure in AAE is often described as a marker of habitual or on-going action rather than as a marker of tense and agreement (Green, 2002; Johnson, 2005). Also, the habitual nature of verbal –*s* has been documented to influence AAE- and/or SWE-speaking children's marking in two studies; however, in both, children's percentages of overt marking have been higher for nonhabitual verbal –*s* forms than for habitual forms (for two interpretations of verbal –*s* in AAE, see Barrière, Kresh, Aharodnik, Legendre, & Nazzi, 2019; Newkirk-Turner & Green, 2016).

We refer to the four probes as dialect-informed because we selected the verbs and manipulated the content before and after the targeted tense and agreement structures to discourage zero marking in AAE (and possibly SWE). Given Hendricks and Adlof's (2017) findings for unmodified and modified scoring approaches and Oetting et al.'s (2016) findings for strategic scoring, we also examined three different scoring approaches: (a) unmodified, which counted only mainstream overt forms; (b) modified, which counted mainstream and nonmainstream overt forms and zero forms; and (c) strategically modified, which counted only mainstream and nonmainstream overt forms and excluded responses that did not obligate the targeted tense and agreement structure.

## Research Questions

In the current work, we asked if dialect-informed probes would reveal tense and agreement deficits in children with SLI in AAE and SWE, and whether the results would vary by the children's dialect, structure targeted, or scoring approach. Based on previous studies with language samples, we expected clinical differences, but we were unsure if the differences would be the same for both dialects, all tense and agreement structures, and all scoring approaches.

## Method

Data were collected between 2010 and 2014. After institutional review board approval, caregiver consent, and child assent, children met with an examiner across multiple sessions at their respective schools. During the first three sessions, standardized tests were administered, and language samples were collected. Then, the four probes for the current study were completed on the following 4 days, with one probe administered each day. The order of the probes was counterbalanced, and as part of these sessions, the children also completed other tasks (e.g., sentence recall, size judgment, nonword repetition; see McDonald & Oetting, 2019; McDonald et al., 2018; Oetting et al., 2016; see also Berry & Oetting, 2017; Gregory & Oetting, 2018; Rivière, Oetting, & Roy, 2018).

### Participants

Participants were 106 children (*M* = 66.24 months, *SD* = 3.78, range: 59–74 months) who lived in a rural area of Louisiana, attended public kindergartens that provided free lunch to all students, and passed a school-based hearing screening. Based on caregiver report, 65 were classified as African American, 34 were classified as Caucasian, one was classified as Asian, one was classified as American Indian, one was classified as mixed race, and four were classified as not reported (with African American reported by the schools for the last five children listed). Each child was classified by dialect as a speaker of either AAE (*n* = 70) or SWE (*n* = 36) and clinical group as either SLI (*n* = 53) or TD (*n* = 53).

The children's AAE (*n* = 70) versus SWE (*n* = 36) dialect classifications corresponded to their African American versus non–African American status. Dialect classifications were initially based on a blind listener judgment task involving three trained listeners who independently rated the children's dialects after listening to 1 min of conversational speech (Oetting & McDonald, 2002). At least two of three listeners rated 90% of the African American children as speakers of AAE and 94% of the non–African American children as speakers of SWE. For those with inconsistent ratings, we then confirmed their dialects using responses from Part I of the Diagnostic Evaluation of Language Variation–Screening Test (Seymour, Roeper, & de Villiers, 2003) and, in a few cases, analysis of longer conversational samples.

The children's clinical group was determined through a battery of standardized tests. Children in both groups passed a hearing screening, scored ≥ −1.2 *SD* of the normative mean on the Primary Test of Nonverbal Intelligence (PTONI; Ehrler & McGhee, 2008), and > −1 *SD* of the normative mean on the Goldman–Fristoe Test of Articulation–Second Edition (GFTA-2; Goldman & Fristoe, 2000). Children in the SLI group scored ≤ −1 *SD* of the normative mean on the syntax portion of the Diagnostic Evaluation of Language Variation–Norm Referenced (DELV–Norm Referenced; Seymour, Roeper, & de Villiers, 2005), whereas those in the TD group scored above this cutoff. For descriptive purposes, the children also completed the Peabody Picture Vocabulary Test–Fourth Edition (PPVT-4; Dunn & Dunn, 2007). Each child with SLI was matched to a TD control based on dialect spoken, age, PTONI, and then, as much as it was possible, maternal educational level.

Summary information about the participants is provided here for convenience, as these same children have been described previously (McDonald & Oetting, 2019; McDonald et al., 2018; Oetting et al., 2016). Table 1 presents the children's ages, maternal education levels, and test scores by their dialect and group. As reported in Oetting et al. (2016), the children's ages did not differ by their dialect or group, but their maternal education levels differed by group (SLI < TD), η_p_^2^ = .05. As expected, the children's PTONI scores did not differ by their dialect or clinical group, and group effects (SLI < TD) were found for the DELV–Norm Referenced, η_p_^2^ = .76. Clinical group effects (SLI < TD) were also observed for the GFTA-2, η_p_^2^ = .15, and PPVT-4, η_p_^2^ = .55, as were dialect effects (AAE < SWE) that were small in magnitude (i.e., GFTA-2, η_p_^2^ = .04; PPVT-4, η_p_^2^ = .05).

**Table 1.** Participant profiles by dialect and clinical status.

Characteristic	AAE		SWE	
	SLI (*n* = 35)	TD (*n* = 35)	SLI (*n* = 18)	TD (*n* = 18)
Age^a^	66.94 (3.74)	65.60 (3.55)	65.72 (3.89)	66.61 (4.18)
Maternal education^b^	11.67 (2.27)	13.27 (2.62)	12.33 (2.87)	13.17 (3.05)
PTONI^c^	93.69 (9.62)	98.09 (8.87)	96.50 (8.35)	98.28 (8.14)
GFTA-2^d^	104.49 (5.72)	107.00 (4.38)	104.78 (4.18)	110.50 (3.09)
DELV–Norm Referenced Syntax^e^	4.83 (1.01)	10.00 (1.55)	4.78 (1.67)	10.39 (1.72)
PPVT-4^f^	82.34 (9.42)	101.06 (9.32)	85.78 (7.01)	105.56 (5.62)

*Note.* Data are reported as mean (standard deviation). Reprinted with modification from Oetting et al. (2016). AAE = African American English; SWE = Southern White English; SLI = children with specific language impairment; TD = typically developing children.

a Age in months.

b Years of schooling (i.e., 12 high school graduates, with data missing for four children).

c Standardized scores for the Primary Test of Nonverbal Intelligence (normative *M* = 100, *SD* = 15).

d Standardized scores for the Goldman–Fristoe Test of Articulation–Second Edition (normative *M* = 100, *SD* = 15).

e Standardized scores for the Syntax portion of the Diagnostic Evaluation of Language Variation–Norm Referenced (normative *M* = 10, *SD* = 3).

f Standardized scores for the Peabody Picture Vocabulary Test–Fourth Edition (normative *M* = 100, *SD* = 15).

### Materials

Four dialect-informed probes were created to elicit 64 tense and agreement structures (i.e., 16 past tense, 16 verbal –*s*, 16 auxiliary BE present, and 16 auxiliary BE past). Each probe included 16 video recordings of puppets and African American and Caucasian adults and children engaged in actions. The recorded actions were 4 s for the past tense, verbal –*s*, and auxiliary BE present probes and 6 s for the auxiliary BE past probe. The actions corresponded to 64 verbs that were selected based on their phonology and ease with which they could be depicted (see Table 2).

**Table 2.** Verbs targeted within the probes.

Past tense	
Regular	dye, fry, mow, play, swallow, tie, tow, show
Irregular	blow, eat, draw, read, ride, tear, throw, write
Verbal –*s*	
Habitual	chew, fly, go, grow, row, saw, sew, spray
Nonhabitual	buy, dry, empty, follow, glue, lay, pay, see
BE present	
Is	clap, fan, make, paint, pound, scratch, stack, stick
Are	bang, cry, drop, punch, open, shiver, sneeze, wash
BE past	
Was	brush, drink, feed, hammer, lick, rock, talk, touch
Were	bounce, bow, build, color, cut, hug, sleep, mix

The past tense probe included 16 items and was designed to elicit eight regular and eight irregular past tense verb forms. To avoid consonant clusters and discourage zero marking, the regular verbs ended with a vowel, liquid, or glide, and all verbs were followed by the article *a* or *an* (e.g., *mowed a lawn*; Pruitt & Oetting, 2009; Rickford, 1999). To encourage past tense responses and following Oetting and Horohov (1997), the children were shown four actions sequentially, with the first two presented in the upper left and right quadrants and the third and fourth presented in the lower left and right quadrants. While each action played, the examiner introduced the target verb in an imperative context (e.g., *Watch the girl dye an egg. Watch her dye an egg. Now she is done*). When the four actions finished, the examiner asked the children to recall the activities while pointing to the stilled actions on the screen and using a prompt that included temporal adverbs (e.g., Now you tell me, “First…., Then…, Then…., Then…”).

The verbal –*s* probe included 16 items and was designed to elicit eight habitual and eight nonhabitual regular verbal –*s* forms. To avoid consonant clusters and discourage zero marking, all verbs for this probe also ended with a vowel, liquid, or glide and were followed by the article *a* or *an*. To encourage present tense and following Seymour et al. (2003), the target verbs were introduced with negative contrast (e.g., *This man doesn*'*t glue a square. He doesn*'*t glue a triangle.* Target response: *He glues a circle*). For each item, the action was played three times, twice while the examiner produced the prompt and once while the children responded. Half of the items were presented with the temporal adverb *always* (e.g., *The man always…*.) to elicit a habitual verbal –*s* form, and the other half were not (e.g., *The man…*) to elicit a nonhabitual verbal –*s* form.

The auxiliary BE present probe included 16 items and was designed to elicit eight *is* and eight *are* auxiliary verb forms. To discourage zero marking, verbs were not constrained by their phonetic content, but nouns, rather than pronouns, served as subjects as this has been shown to make a difference (Garrity & Oetting, 2010; Wyatt, 1991). For each item, the examiner showed the action in a still frame and provided an introductory prompt (e.g., *The mouse seems strong. Tell me what you see*). Then, the examiner played the action to elicit the child's response (e.g., *The mouse is pushing a car*).

The auxiliary BE past probe included 16 items and was designed to elicit eight *was* and eight *were* auxiliary forms. Again, to discourage zero marking, verbs were not constrained by their phonetic content, but nouns, rather than pronouns, served as subjects. Before each video, the examiner introduced the action using a verb in an imperative context (e.g., *Watch the bear touch his ears*). Then, the examiner played the action and repeated the prompt two more times. While the action played for the third time, the examiner covered the screen and asked the children what they remembered seeing (e.g., Examiner: *Before I covered this up, tell me what you remember the bear doing*; Child: *The bear was touching his ears*).

Prior to each probe, four additional verbs and video recordings of actions were used for training. Administration of the probes was audio-recorded for later coding.

### Coding

Coders were unaware of the children's dialect and clinical group. Items were coded based on the full response produced by the child, which occasionally resulted in a child producing a different verb in the same subcategory intended by the probe (e.g., child produced irregular *drive/drove* for the target irregular *ride/rode*); these responses were coded if they were not repetitions of a child's earlier response. On other rare occasions, a child's response resulted in an item falling into a different subcategory; these responses were also coded if they were not repetitions. For example, if a child produced a past tense regular verb for a past irregular item, the child's response was coded as a regular item. Similarly, if a child produced the word *always* with a nonhabitual verbal –*s* item or a singular subject for a plural BE item, the child's response was coded as a habitual or singular BE item, respectively. Finally, the children's *is* and *was* responses were included in calculations of *are* and *were* if the grammatical subject was plural (e.g., *The bears is/was…*), and *are* and *were* forms were included in calculations of *is* and *was* if the grammatical subject was singular *(e.g., The bear are/were…*). This coding decision is consistent with standard test administration practices but differs from what has been done in other AAE and SWE studies, which have coded children's BE forms based on surface structure (e.g., coding a response as *was* regardless of the subject; Berry & Oetting, 2017; Newkirk-Turner, Oetting, & Stockman, 2014, 2016; Oetting & Garrity, 2006; Roy, Oetting, & Moland, 2013).

The children's responses were coded by type (i.e., Mainstream Overt, Nonmainstream Overt, Zero, Other) and, if applicable, type of nonmainstream overt form (e.g., double regular marking, overregularization, *had + verb*, *is* for *are*, *was* for *were*). Zero marked forms required a child's response to include a subject (e.g., *the boy, he*), and for the auxiliary BE probes, a verb with progressive marking (e.g., *mixing*) to ensure that the child was producing a predicate that obligated the target structure. Responses classified as Other often related to the item but did not obligate the target structure (e.g., target: *The lady was brushing a horse*, child response: *I like to brush horses*, *brush, brushed*, *brushing, is brushing*). In addition, responses elicited for the past tense and verbal –*s* probes were coded for following context (i.e., consonant vs. nonconsonant), and those elicited for the auxiliary BE probes were coded for preceding subject context (i.e., noun vs. personal pronoun) so we could check for potential context effects on form production. Finally, 57 (< 1%) of the 6,784 (64 items × 106 children) responses were excluded from the analyses. These included 26 involving a child's repetition of a previously coded response, 16 that did not elicit a response from a child or the response was accompanied by noise on the recording that precluded scoring, and 15 with an accidental examiner model of the target that was followed by a child producing an overtly marked form.^3^ The average number of responses coded per participant was 63.46 (*SD* = 0.84, range: 61–64), and for each probe, was the average number of responses coded per participant: 15.85 for past tense (*SD* = 0.38, range: 14–16), 15.94 for verbal –*s* (*SD* = 0.27, range: 14–16), 15.81 for BE present (*SD* = 0.46, range: 14–16), and 15.86 for BE past (*SD* = 0.47, range: 13–16).

### Reliability of Coding

Reliability was examined yearly by having a second examiner independently code 20% of all probes collected. Coding agreement for each probe ranged from 94% to 97% and was above 90% every year; across probes and years, agreement was 95%.

### Scoring Approaches

#### Unmodified

The unmodified scoring approach was consistent with Hendricks and Adlof's (2017) unmodified approach, and it was designed to mirror what is typically scored on a test designed for GAE speakers. For this approach, only Mainstream Overt forms of the targeted tense and agreement structures were scored as overtly marked. All Nonmainstream Overt, Zero, and Other responses were scored as errors. This approach penalizes children who produce nonmainstream forms of AAE and SWE.

#### Modified

The modified scoring approach was consistent with Hendricks and Adlof's (2017) modified approach, and it was designed to mirror what is typically recommended to accommodate children's dialect differences when scoring a test designed for GAE speakers. For this approach, all Mainstream and Nonmainstream Overt forms and Zero forms of AAE and SWE were counted as dialect appropriate. The children's Other responses were scored as errors. Although this approach does not penalize children who produce nonmainstream forms, it does not allow for the detection of clinical group differences in children's overt marking of tense and agreement. Instead, it relies on differences in the responses classified as Other to differentiate the clinical groups.

#### Strategic

The strategic scoring approach was designed to capture the specific tense and agreement profile of children with SLI in AAE, SWE, GAE, and other languages. This scoring approach considered all Mainstream and Nonmainstream Overt forms as marked and all Zero forms as unmarked. Although Zero forms are allowable in AAE and SWE, they were scored as unmarked because we expected the SLI groups to produce higher percentages of these forms than the TD controls (for a similar scoring approach, see Berry & Oetting, 2017; Garrity & Oetting, 2010; Newkirk-Turner et al., 2014; Pruitt & Oetting, 2009; Roy et al., 2013). In addition, all responses classified as Other were excluded from the strategic scoring approach. Excluding these responses is consistent with the scoring of tense and agreement productivity as operationalized within the Rice/Wexler Test of Early Grammatical Impairment (Rice & Wexler, 2001). As noted by Rice and Wexler (2001), children with SLI primarily produce tense and agreement errors of omission (e.g., target verbal –*s*: *Today, he walkØ*) rather than errors of commission (e.g., target verbal –*s*: *Today, he can walks*; for additional support of this claim, see Cleave & Rice, 1997; Eadie, Fey, Douglas, & Parsons, 2002; Leonard, Bortolini, Caselli, McGregor, & Sabbadini, 1992; Rice, 2003, 2009; Rice & Blossom, 2013; Rice & Wexler, 1996; Rice, Wexler, & Cleave, 1995).

It is also important to note that the current strategic scoring approach differs from the one implemented by Oetting et al. (2016) for sentence recall, which counted as correct verbal –*s* zero forms in addition to two nonmainstream overt forms (i.e., *is* for *are*, *was* for *were*). For now, we note the scoring differences across studies and consider these differences within the discussion.

## Results

### Preliminary Analyses

#### Linguistic Context Effects

To discourage zero marking, the past tense and verbal –*s* probes were designed to elicit a nonconsonant (i.e., *a* or *an*) as a following context and the BE probes were designed to elicit a noun for the preceding subject context. The probes varied in their success to elicit the intended contexts; 66% of the elicited past tense forms were followed by nonconsonant compared to 84% of the verbal –*s* forms, and 42% of the elicited auxiliary BE present forms were preceded by a noun subject as compared to 16% of the auxiliary BE past forms. Nevertheless, when tested with four 2 (clinical group) × 2 (dialect) × 2 (context) analyses of variance (ANOVAs), the children's percentages of zero marked forms did not vary by type of following or preceding context (all *p*s > .20 and all η_p_^2^s ≤ .06).

#### School Enrollment

We also examined school enrollment as a possible influence on the children's zero marking. In an earlier study of these children and others from the same schools, Riviére et al. (2018) found higher percentages of zero marked infinitival TO (e.g., *Aaron wants Ø eat*) for children enrolled in one of the schools as compared to the others, and they tied this to the Cajun heritage of the children's school and community. Given that elderly Cajun English speakers also zero mark tense and agreement structures (Dubois & Horvath, 2003), it was important to check for school effects within the children's zero marking of these structures. Results indicated that, for all four probes, percentages of zero marking for the school that celebrated its Cajun heritage were not statistically higher than those of the other schools (all *p*s > .43 and all η_p_^2^s ≤ .001). These findings are consistent with Oetting and Garrity (2006), who also did not find a Cajun status effect when examining children's marking of tense and agreement in a neighboring community. Results from the preliminary analyses indicated that we could examine the children's marking of tense and agreement without being concerned about following or preceding context effects or school effects.

### Probes Combined

**Figure 1.**
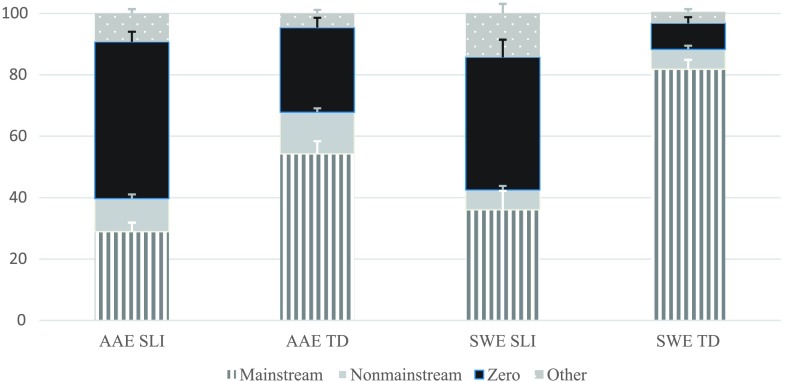
Probes combined: response types by dialect and group. AAE SLI = African American English–speaking children with specific language impairment; AAE TD = African American English–speaking children with typical development; SWE SLI = Southern White English–speaking children with specific language impairment; SWE TD = Southern White English–speaking children with typical development.

The children's marking of tense and agreement was first examined by combining the four probes. As shown in Figure 1, all four groups produced Mainstream Overt, Nonmainstream Overt, Zero, and Other responses; however, the TD groups produced the highest proportions of Mainstream Overt responses, and the SLI groups produced the highest proportions of Zero forms and Other responses. In addition, the AAE TD group produced the highest proportion of Nonmainstream Overt responses, followed by the AAE SLI group.

Using these responses and the three scoring approaches, percentages of marking were calculated. As shown in Table 3, percentages were lowest with unmodified scoring and highest with modified scoring, with the latter averaging > 90% for three of the four groups. The strategic approach led to percentages that fell in the middle of the others. When each scoring approach was examined with a 2 (clinical group) × 2 (dialect) ANOVA, all three approaches led to group differences (SLI < TD); however, unmodified scoring yielded a dialect by group interaction that reflected a larger effect size of the difference in SWE (η_p_^2^ = .56) than in AAE (η_p_^2^ = .27). The other two approaches yielded main effects for group, with strategic scoring showing a larger effect size of the difference than modified scoring (strategic: SLI *M* = 45, *SD* = 25 < TD *M* = 77, *SD* = 20, η_p_^2^ = .38 vs. modified: SLI *M* = 89, *SD* = 10 < TD *M* = 96, *SD* = 06, η_p_^2^ = .17). Thus, both strategic and modified scoring leveled differences between the two dialects and still showed clinical group differences, but strategic was better at the latter.

**Table 3.** Probes combined: mean (standard deviation) percent marking by scoring approach, dialect, and clinical group.

Approach	AAE		SWE	
	SLI	TD	SLI	TD
Unmodified	29 (17)	54 (24)	36 (26)	82 (13)
Strategic	43 (22)	71 (20)	48 (30)	91 (10)
Modified	91 (08)	95 (07)	86 (13)	97 (04)
Significant effects				
Unmodified	Group, *F*(1, 102) = 68.81, *p* < .001, η_p_^2^ = .40			
	Dialect, *F*(1, 102) = 16.33, *p* < .001, η_p_^2^ = .14			
	Group × Dialect, *F*(1, 102) = 5.72, *p* = .019, η_p_^2^ = .05			
	TD dialect, *F*(1, 51) = 20.05, *p* < .001, η_p_^2^ = .28			
	AAE group, *F*(1, 68) = 25.53, *p* < .001, η_p_^2^ = .27			
	SWE group, *F*(1, 34) = 43.69, *p* < .001, η_p_^2^ = .56			
Strategic	Group, *F*(1, 102) = 63.62, *p* < .001, η_p_^2^ = .38			
	Dialect, *F*(1, 102) = 7.82, *p* = .006, η_p_^2^ = .07			
Modified	Group, *F*(1, 102) = 20.53, *p* < .001, η_p_^2^ = .17			
Classification accuracy SLI vs. TD				
Unmodified	Cut score = 47%, classification accuracy 75%, Se = .81, Sp = .68			
Strategic	Cut score = 61%, classification accuracy 75%, Se = .72, Sp = .77			
Modified	Cut score = 93%, classification accuracy 66%, Se = .51, Sp = .81			

*Note.* AAE = African American English; SWE = Southern White English; SLI = children with specific language impairment; TD = typically developing children.

We then examined the classification accuracy (SLI vs. TD) of the three scoring approaches using discriminant analyses. As also shown in Table 3, the unmodified and strategic approaches yielded a classification accuracy rate of 75%, with unmodified having a high level of sensitivity (.81) and low level of specificity (.68) and strategic having relative balanced levels of sensitivity and specificity (.72 and .77, respectively). In comparison to these two approaches, the modified approach yielded a lower overall classification accuracy of 66%, with a low level of sensitivity (.51) and high level of specificity (.81).

### Probes Separated

**Figure 2.**
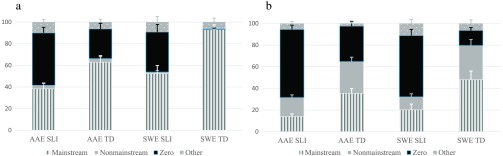
(a) Past tense regular responses: response types by dialect and group. (b) Past tense irregular responses: response types by dialect and group. AAE SLI = African American English–speaking children with specific language impairment; AAE TD = African American English–speaking children with typical development; SWE SLI = Southern White English–speaking children with specific language impairment; SWE TD = Southern White English–speaking children with typical development.

As shown in Figures 2 [Fig F3] [Fig F4]–5, proportions of Mainstream Overt, Nonmainstream Overt, Zero, and Other responses varied by the children's dialect and clinical group and by the probe and structure examined. As with the probes combined, we first describe the children's responses for each probe. Then, we present percentages of marking using the three scoring approaches and analyze these percentages with a series of 2 (clinical status) × 2 (dialect) × 2 (structure) ANOVAs (see Tables 4 [Table T5] [Table T6]–7). Each analysis included 106 children except for the strategically scored auxiliary BE past probe, which included 101 children; the five children excluded from this analysis were two with SLI (one AAE, one SWE) who did not produce any responses obligating a *was* and *were* form and three with SLI (one AAE, two SWE) who did not produce any responses obligating a *was* form.

**Figure 3. F3:**
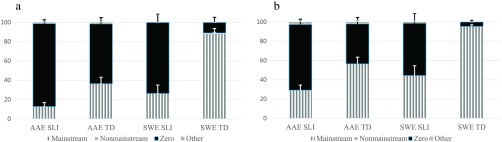
(a) Verbal –*s* habitual responses: response types by dialect and group. (b) Verbal –*s* nonhabitual responses: response types by dialect and group. AAE SLI = African American English–speaking children with specific language impairment; AAE TD = African American English–speaking children with typical development; SWE SLI = Southern White English–speaking children with specific language impairment; SWE TD = Southern White English–speaking children with typical development.

**Figure 4. F4:**
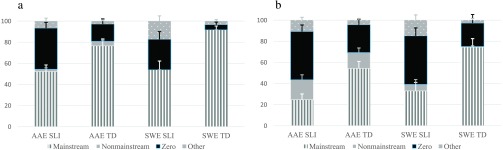
(a) BE present *is* responses: response types by dialect and group. (b) BE present *are* responses: response types by dialect and group. AAE SLI = African American English–speaking children with specific language impairment; AAE TD = African American English–speaking children with typical development; SWE SLI = Southern White English–speaking children with specific language impairment; SWE TD = Southern White English–speaking children with typical development.

**Figure 5.**
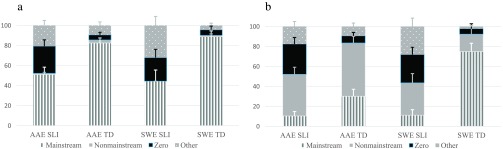
(a) BE past *was* responses: response types by dialect and group. (b) BE past *were* responses: response types by dialect and group. AAE SLI = African American English–speaking children with specific language impairment; AAE TD = African American English–speaking children with typical development; SWE SLI = Southern White English–speaking children with specific language impairment; SWE TD = Southern White English–speaking children with typical development.

**Table 4.** Past tense: mean (standard deviation) percent marking by scoring approach, dialect, and group.

Approach	AAE		SWE	
	SLI	TD	SLI	TD
Regular				
Unmodified	38 (31)	63 (36)	52 (31)	93 (18)
Strategic	46 (31)	70 (33)	57 (33)	98 (8)
Modified	90 (13)	94 (15)	91 (22)	94 (16)
Irregular				
Unmodified	14 (14)	35 (26)	20 (23)	48 (34)
Strategic	34 (22)	66 (29)	35 (26)	85 (12)
Modified	95 (11)	98 (07)	89 (15)	94 (14)
Significant effects				
Unmodified	Group, *F*(1, 102) = 37.74, *p* < .001, η_p_^2^ = .27			
	Dialect, *F*(1, 102) = 11.38, *p* = .001, η_p_^2^ = .10			
	Structure, *F*(1, 102) = 100.69, *p* < .001, η_p_^2^ = .50			
	Dialect × Structure, *F*(1, 102) = 3.95, *p* = .04, η_p_^2^ = .04			
	AAE structure, *F*(1, 68) = 44.86, *p* < .001, η_p_^2^ = .40			
	SWE structure, *F*(1, 34) = 62.52, *p* < .001, η_p_^2^ = .65			
	Regular dialect, *F*(1, 102) = 12.02, *p* = .001, η_p_^2^ = .11			
Strategic	Group, *F*(1, 102) = 54.33, *p* < .001, η_p_^2^ = .35			
	Dialect, *F*(1, 102) = 9.20, *p* = .003, η_p_^2^ = .08			
	Structure, *F*(1, 102) = 27.12, *p* < .001, η_p_^2^ = .21			
Modified	Null findings			

*Note.* AAE = African American English; SWE = Southern White English; SLI = children with specific language impairment; TD = typically developing children.

**Table 5. T5:** Verbal –*s*: mean (standard deviation) percent marking by scoring approach, dialect, and group.

Approach	AAE		SWE	
	SLI	TD	SLI	TD
Habitual				
Unmodified	13 (24)	36 (40)	26 (37)	88 (22)
Strategic	13 (24)	36 (40)	26 (37)	89 (23)
Modified	99 (4)	99 (5)	100 (0)	100 (0)
Nonhabitual				
Unmodified	29 (32)	57 (41)	44 (43)	95 (7)
Strategic	30 (32)	57 (41)	45 (43)	95 (7)
Modified	97 (8)	98 (9)	99 (6)	100 (0)
Significant effects				
Unmodified	Group, *F*(1, 102) = 40.72, *p* < .001, η_p_^2^ = .29			
	Dialect, *F*(1, 102) = 21.58, *p* < .001, η_p_^2^ = .18			
	Structure, *F*(1, 102) = 39.99, *p* < .001, η_p_^2^ = .28			
	Group × Dialect, *F*(1, 102) = 5.84, *p* = .017, η_p_^2^ = .05			
	AAE group, *F*(1, 68) = 10.83, *p* = .002, η_p_^2^ = .14			
	SWE group, *F*(1, 34) = 34.00, *p* < .001, η_p_^2^ = .50			
	TD dialect, *F*(1, 51) = 22.90, *p* < .001, η_p_^2^ = .32			
Strategic	Group, *F*(1, 102) = 40.56, *p* < .001, η_p_^2^ = .29			
	Dialect, *F*(1, 102) = 21.59, *p* < .001, η_p_^2^ = .18			
	Structure, *F*(1, 102) = 39.74, *p* < .001, η_p_^2^ = .28			
	Group × Dialect, *F*(1,102) = 5.99, *p* = .016, η_p_^2^ = .06			
	AAE group, *F*(1, 68) = 10.58, *p* = .002, η_p_^2^ = .14			
	SWE group, F(1, 34) = 34.28, *p* < .001, η_p_^2^ = .50			
	TD dialect, *F*(1, 51) = 24.17, *p* < .001, η_p_^2^ = .32			
Modified	Null findings			

*Note.* AAE = African American English; SWE = Southern White English; SLI = children with specific language impairment; TD = typically developing children.

**Table 6. T6:** BE present: mean (standard deviation) percent marking by scoring approach, dialect, and group.

Approach	AAE		SWE	
	SLI	TD	SLI	TD
Is				
Unmodified	52 (37)	76 (33)	54 (35)	92 (15)
Strategic	57 (36)	83 (30)	63 (38)	94 (12)
Modified	93 (16)	97 (6)	83 (21)	97 (6)
Are				
Unmodified	25 (32)	54 (39)	33 (34)	74 (35)
Strategic	49 (38)	72 (34)	43 (36)	77 (35)
Modified	89 (16)	96 (10)	85 (21)	97 (7)
Significant effects				
Unmodified	Group, *F*(1, 102) = 31.97, *p* < .001, η_p_^2^ = .24			
	Structure, *F*(1, 102) = 33.69, *p* < .001, η_p_^2^ = .25			
Strategic	Group, *F*(1, 102) = 20.29, *p* < .001, η_p_^2^ = .17			
	Structure, *F*(1, 102) = 23.29, *p* < .001, η_p_^2^ = .19			
Modified	Group, *F*(1, 102) = 12.42, *p* < .001, η_p_^2^ = .11			

*Note.* AAE = African American English; SWE = Southern White English; SLI = children with specific language impairment; TD = typically developing children.

**Table 7.** BE past: mean (standard deviation) percent marking by scoring approach, dialect, and group.

Approach	AAE		SWE	
	SLI	TD	SLI	TD
Was				
Unmodified	51 (43)	83 (29)	44 (48)	89 (25)
Strategic	64 (44)	92 (24)	59 (46)	92 (24)
Modified	79 (30)	91 (21)	68 (38)	96 (10)
Were				
Unmodified	10 (26)	30 (41)	11 (23)	75 (35)
Strategic	59 (45)	91 (25)	57 (42)	94 (24)
Modified	83 (30)	91 (20)	72 (36)	98 (5)
Significant effects				
Unmodified	Group, *F*(1, 102) = 50.03, *p* < .001, η_p_^2^ = .33			
	Dialect, *F*(1, 102) = 4.00, *p* = .048, η_p_^2^ = .04			
	Structure, *F*(1, 102) = 62.05, *p* < .001, η_p_^2^ = .38			
	Group × Dialect, *F*(1, 102) = 6.43, *p* = .013, η_p_^2^ = .06			
	AAE group, *F*(1, 68) = 15.20, *p* < .001, η_p_^2^ = .18			
	SWE group, *F*(1, 34) = 34.76, *p* < .001, η_p_^2^ = .51			
	TD dialect, *F*(1, 51) = 11.72, *p* < .001, η_p_^2^ = .19			
	Dialect × Structure, *F*(1, 102) = 6.75, *p* = .01, η_p_^2^ = .06			
	AAE structure, *F*(1, 68) = 74.80, *p* < .001, η_p_^2^ = .52			
	SWE structure, *F*(1, 34) = 12.56, *p* = .001, η_p_^2^ = .27			
	Were dialect, *F*(1, 102) = 11.44, *p* = .001, η_p_^2^ = .10			
Strategic	Group, *F*(1, 97) = 20.57, *p* < .001, η_p_^2^ = .18			
Modified	Group, *F*(1, 102) = 12.78, *p* = .001, η_p_^2^ = .11			

*Note.* AAE = African American English; SWE = Southern White English; SLI = children with specific language impairment; TD = typically developing children.

#### Past Tense

As shown in Figures 2a and 2b, the highest proportions of Mainstream Overt forms were produced for the regular items, and the highest proportions of Nonmainstream Overt forms were produced for the irregular items. Of the Nonmainstream Overt forms, most involved overregularizations (e.g., *eated, drived*), with the AAE groups also producing a small number of *had + verb* forms (AAE SLI *n* = 9, AAE TD *n* = 16). As was found for the probes combined, the TD groups produced the highest proportions of Mainstream and Nonmainstream Overt forms, and the SLI groups produced the highest proportions of Zero forms and Other responses.

As shown in Table 4, unmodified scoring of the past tense probe yielded main effects for clinical group (SLI *M* = 29, *SD* = 21 < TD *M* = 56, *SD* = 26), dialect and structure, and an interaction between dialect and structure, which was related to a dialect effect for regular items (AAE *M* = 51, *SD* = 35 < SWE *M* = 73, *SD* = 33, η_p_^2^ = .09), but not irregular items. Strategic scoring also yielded a main effect for group (SLI *M* = 41, *SD* = 25 < TD *M* = 76, *SD* = 25), in addition to main effects for structure (regular *M* = 64, *SD* = 35 > irregular *M* = 53, *SD* = 31) and dialect (AAE *M* = 53, *SD* = 29 < SWE *M* = 69, *SD* = 31). In comparing these two approaches, the effect size of the group difference was slightly larger with strategic scoring than with unmodified scoring (η_p_^2^ = .35 vs. .27). In contrast to these two approaches, modified scoring yielded no group, dialect, or structure effects; all groups were essentially at ceiling.

#### Verbal –s

As shown in Figures 3a and 3b, the highest proportions of Mainstream Overt forms were produced for the nonhabitual items, and the highest proportions of Zero forms were produced for the habitual items. In addition, the TD groups produced the highest proportions of Mainstream Overt forms, and the SLI groups produced the highest proportions of Zero forms. In contrast to the other probes, nonmainstream overt forms were extremely rare. In fact, only one SWE TD child produced one Nonmainstream Overt form, which involved double marking of verbal –*s* on the verb *row*.^4^ Responses classified as Other were also infrequent.

Given the limited number of other responses on the verbal –*s* probe, the ANOVA results for unmodified and strategic scoring were nearly identical (see Table 5). Both scoring approaches yielded group and dialect main effects, which were qualified by interactions between group and dialect. Follow-up of the interactions showed a group effect (SLI < TD) in both dialects, but the effect size was larger in SWE (η_p_^2^ = .50) than in AAE (η_p_^2^ = .14). In addition, a dialect effect was observed for the TD group (η_p_^2^ = .32), but not the SLI group. Both scoring approaches also yielded a main effect for structure (unmodified: habitual *M* = 36, *SD* = 41 < nonhabitual *M* = 52, *SD* = 41; strategic: habitual *M* = 36, *SD* = 41 < nonhabitual *M* = 53, *SD* = 41). In contrast to these two approaches, modified scoring yielded no group, dialect, or structure effects as again performance was at ceiling across the board.

#### Auxiliary BE Present

As shown in Figures 4a and 4b, the highest proportions of Mainstream Overt forms were produced for the *is* items, and the highest proportions of Nonmainstream Overt forms and Zero forms were produced for the *are* items. In addition, the SWE TD group produced the highest proportion of Mainstream Overt forms, and the AAE speakers, regardless of their clinical status, produced the highest proportions of Nonmainstream Overt forms, with most of these reflecting the children's use of *is* with plural subjects (e.g., *The puppets is…, They is, They*'*s…*). Like the other probes, the SLI groups produced the highest proportions of Zero forms and Other responses.

As shown in Table 6, unmodified and strategic scoring of this probe yielded main effects for group (unmodified: SLI *M* = 40, *SD* = 29 < TD *M* = 71, *SD* = 29; strategic: SLI *M* = 53, *SD* = 34 < TD *M* = 80, *SD* = 27) and structure (unmodified: *is M* = 67, *SD* = 36 > *are M* = 44, *SD* = 40; strategic: *is M* = 73, *SD* = 35 > *are M* = 60, *SD* = 38). Modified scoring also yielded a main effect for group (SLI *M* = 89, *SD* = 17 < TD *M* = 97, *SD* = 06), but the effect size was smaller than those generated by the other two approaches (modified: η_p_^2^ = .11 vs. unmodified: η_p_^2^ = .24 and strategic: η_p_^2^ = .17). The modified scoring approach also yielded no structure effects, and there were no dialect effects with any of the scoring approaches.

#### Auxiliary BE Past

As shown in Figures 5a and 5b, the highest proportions of Mainstream Overt forms were produced for the *was* items, and the highest proportions of Nonmainstream Overt forms were produced for the *were* items (and all of these involved the children's use of *was* as in *The bears was…*). In addition, the SWE TD group produced the highest proportion of Mainstream Overt forms, the AAE TD group produced the highest proportion of Nonmainstream Overt forms, and the SLI groups produced the highest proportions of Zero forms and Other responses. Compared to the other probes, the auxiliary BE past probe elicited the highest proportions of Nonmainstream Overt forms and Other responses.

Unmodified scoring yielded group and dialect main effects that interacted (see Table 7). Like what was found for the past tense probe, the interaction related to a group effect (SLI < TD) in both dialects, but the effect size was larger in SWE (η_p_^2^ = .51) than in AAE (η_p_^2^ = .18). In addition, a dialect effect was observed for the TD group (η_p_^2^ = .19), but not the SLI group. The unmodified approach also yielded an interaction between dialect and structure that related to a structure effect in both dialects, but with a larger effect size in AAE (*was M* = 67, *SD* = 40 > *were M* = 20, *SD* = 36, η_p_^2^ = .52) than in SWE (*was M* = 67, *SD* = 44 > *were M* = 43, *SD* = 44, η_p_^2^ = .27), and a dialect effect for *were* (AAE *M* = 20, *SD* = 36 < SWE M 43, *SD* = 44), but not *was*. In comparison to the unmodified approach, both strategic and modified scoring yielded a group main effect (strategic: SLI *M* = 57, *SD* = 44 < TD *M* = 92, *SD* = 22; modified: SLI *M* = 77, *SD* = 31 < TD *M* = 93, *SD* = 17) without effects for dialect or structure. Although these approaches yielded group differences, the effect size of the difference was larger with strategic scoring than with modified scoring (η_p_^2^ = .18 vs. .11).

In summary, with unmodified and strategic scoring, the ANOVA results repeatedly showed clinical group differences (SLI < TD), while also showing some structure, dialect, and scoring differences depending on the probe examined. As was found for the probes combined, the strategic scoring approach was superior to the others because it led to the fewest number of interactions between the children's clinical groups and dialects and between the individual structures and the children's dialects. Although the modified approach was designed to reduce dialect differences (and no dialect differences were observed), this approach was the least successful for identifying differences between the groups. We now turn to a series of exploratory discriminant function analyses using the strategic scoring approach.

### Classification Accuracy of the Tense and Agreement Probes Using Strategic Scoring

Using percentages of overt marking from the four probes and eight structures, we ran multiple discriminant function analyses. These were run with the dialects combined and separated, and they included 106 children except for analyses with *was* and *were*, which included 101 or 104 children, respectively. We report the three most successful analyses below.

For all eight structures treated as separate variables, classification accuracy was 78% (Se = .71 and Sp = .85), but this did not include the five excluded children with SLI. Divided into dialects, classification accuracy in AAE and SWE was 77% (Se = .70 and Sp = .83) and 94% (Se = .93 and Sp = .94), respectively. Of the four probes, past tense led to the highest classification levels. With the dialects combined and using a cut score of .60, the overall accuracy was 77% (Se = .74 and Sp = .81). Divided by dialects and using cut scores of .54 and .73, respectively, classification accuracy in AAE and SWE was 71% (Se = .71, Sp = .71) and 86% (Se = .72 and Sp = 1.00). Finally, of the eight structures, past tense irregular led to the highest classification levels. For the dialects combined and with a cut score of .56, the overall accuracy was 79% (Se = .79, Sp = .79). Divided by dialects and using cut scores of .50 and .71, respectively, accuracy in AAE and SWE was 76% (Se = 74%, Sp = 77%) and 89% (Se = .89, Sp = .89). As evident across all three discriminant analyses presented (and all analyses we attempted), different cut scores were identified as optimal when we examined the two dialects separately, meaning that there is no universal standard for tense and agreement marking against which speakers of various English dialects can or should be compared. In addition, for all analyses completed, there were dialect differences which yielded lower classification accuracy levels in AAE than in SWE.

## Discussion

GAE-speaking children with SLI overtly mark tense and agreement at lower percentages than same dialect–speaking TD controls, and studies of language samples report a similar finding within AAE and SWE for at least some tense and agreement structures. In the current work, we asked if dialect-informed probes would also reveal tense and agreement deficits in AAE- and SWE-speaking children with SLI, and whether the results would vary by the children's dialect, structure targeted, and/or scoring approach. Answers to both questions were yes.

With the probes combined, all three scoring approaches showed the children with SLI to overtly mark tense and agreement at lower percentages than the TD controls; however, strategic scoring was superior to the others because it led to a group difference that did not interact with the children's dialects and an effect size of the group difference that was twice as large as the effect with modified scoring (η_p_^2^ = .38 vs. .17). Of the three approaches, strategic scoring also had the most balanced levels of sensitivity (.72) and specificity (.77). Unmodified scoring led to a low level of specificity (which indicates overidentification of children as SLI), and modified scoring led to a low level of sensitivity (which indicates underidentification); these findings for unmodified and modified scoring replicate those reported by Hendricks and Adlof (2017) for the CELF-5.

When examining the probes individually, unmodified and strategic scoring continued to show clinical group differences. In addition, there were some interactions of group and dialect with unmodified scoring (verbal –*s* and BE past) and with strategic scoring (verbal –*s*), and these interactions indicated stronger clinical effects in SWE than in AAE. Therefore, these two scoring approaches did not level all dialect differences, but they allowed for clinical group differences to be detected. Modified scoring also led to group differences for BE present and BE past, but as was found for the probes combined, this scoring approach led to smaller effect sizes of the clinical differences than the other scoring approaches.

Using strategic scoring, the highest overall levels of classification accuracy (SLI vs. TD) were found for all eight structures considered as separate variables (78%), the past tense probe (77%), and the past tense probe irregular items (79%). Divided by dialects, different cut scores were identified as optimal for differentiating the SLI and TD groups; however, all analyses showed dialect differences, with lower accuracy levels in AAE than in SWE.

### Clinical Implications

The findings provide strong evidence that tense and agreement deficits are a component of the SLI profile in AAE and SWE and indicate that these structures should be assessed and treated when working with AAE- and SWE-speaking children with SLI. That said, there are multiple ways to approach the scoring of these structures and multiple ways to use tense and agreement data within clinical practice. Regarding scoring approaches, the strategic approach was deemed most useful, and the modified approach was deemed least useful. Regarding clinical practice, the findings indicate that strategic scoring of the past tense probe (and the children's overt marking of past tense irregular items within this probe) is most useful for identifying children with SLI in AAE and SWE. These finding are consistent with others who have identified past tense marking (either regular, irregular, or both) as diagnostically useful in SLI studies of children learning GAE, AAE, SWE, and English as a second language (e.g., Blom & Paradis, 2013; Jacobson & Livert, 2010; Jacobson & Schwartz, 2005; Oetting & Garrity, 2006; Oetting & Horohov, 1997; Rice, Wexler, Marquis, & Hershberger, 2000; Seymour et al., 1998; Weiler, Schuele, Feldman, & Krimm, 2018; Werfel, Hendricks, & Schuele, 2017; see also Krok & Leonard, 2015). Unfortunately, even with strategic scoring, the classification accuracies of the probes, including the past tense probe, were lower in AAE than in SWE and lower than 90% with the dialects combined and separated. Given that 90% overall accuracy (with Se and Sp also ≥ .90) is often now considered the gold standard within assessment (Dollaghan, 2007), it would be unwise to base clinical decisions regarding a child's SLI status on these probes alone. But a speech-language pathologist would never rely on a single set of grammar measures for determining a diagnosis, so this finding is not inconsistent with current best practice within the field. More importantly, the large effect sizes generated by the current tense and agreement probes demonstrate their potential when combined with other assessment data (e.g., teacher, parent, and child questionnaires and receptive and expressive measures of vocabulary, complex syntax, narratives, pragmatics, and preliteracy/literacy).

Also, there are additional goals of assessment beyond arriving at a diagnosis, and these include describing the strengths and weaknesses of a child's language system and securing baseline data for goal writing and progress monitoring. For these assessment goals, the current probes would be extremely useful. Tense and agreement structures play a key role in the utterances children produce when conversing with family, friends, and teachers, and they are found in the sentences children hear, read, and write in and outside school. Although language samples can be used to collect data from children, they are time consuming to elicit and transcribe, and they often lack high numbers of tense and agreement contexts for analysis. The current probes not only led to more data than are traditionally elicited with samples, but they also allowed us to manipulate in dialect-informed ways the verbs selected and the content that preceded and followed the targeted tense and agreement structure. Finally, administration of each probe took no longer than 10 min, and the children's responses were coded without transcribing the sessions. That the strategically scored probes led to clinical group differences with large effects sizes (η_p_^2^ ranged from .14 to .50, with corresponding Cohen's *d* ranging from 0.77 to 1.85) demonstrates their usefulness for eliciting high numbers of diverse and diagnostically relevant tense and agreement forms from AAE- and SWE-speaking children.

We also would argue that, although a goal of nonbiased test development is often to remove dialect differences (and the DELV–Norm Referenced is an example of a test that has done just that), some measures within the profession should help a clinician understand a child's dialect. The current probes provide a clinician a mechanism for achieving this goal. Recall that the AAE- and SWE-speaking children studied here produced mainstream and nonmainstream overt forms and zero forms when expressing tense and agreement, and most of their nonmainstream overt forms included overregularizations, preterite *had + verbs*, and use of *is* and *was* with plural subjects. In addition, the AAE speakers produced a higher number and a more diverse range of nonmainstream overt forms and zero forms than the SWE speakers. These findings are highly consistent with the adult and child AAE and SWE dialect literature as are the dialect-specific structure effects that were documented. Using the strategically scored results to illustrate these latter effects, recall that the AAE TD group overtly marked past tense (regular 70%, irregular 66%) at higher percentages than verbal –*s* (habitual 36%, nonhabitual 57%) and auxiliary BE past (*was* 92%, *were* 91%) at higher percentages than auxiliary BE present (*is* 83%, *are* 72%) and that the SWE TD group overtly marked all structures at relatively high percentages (i.e., past regular 98%, past irregular 85%, verbal –*s* habitual 89%, verbal –*s* nonhabitual 95%, auxiliary *is* 94%, *was* 92%, *were* 94%), except for auxiliary *are*, which was overtly marked at a lower percentage (77%). Recall also (and as just shown), both dialect groups overtly marked nonhabitual verbal –*s* (AAE 57%, SWE 95%) at higher percentages than habitual verbal –*s* (AAE 36%, SWE 89%), and this finding replicates two other dialect studies (Cleveland & Oetting, 2013; Newkirk-Turner & Green, 2016). As clinicians, we would want to know this dialect information about the children and communities we serve. To illustrate the importance of this type of information, Berry and Oetting (2017) administered the current auxiliary BE probes to a group of AAE-speaking TD children with Gullah Geechee heritage, and the results showed both qualitative and quantitative influences of Gullah Geechee within the children's responses. This auxiliary BE data can now be showcased and contrasted with data from AAE speakers to celebrate and preserve the languages, dialects, and cultures of the Gullah Geechee Corridor (e.g., Berry, 2018; McLeod, 2018).

As experts of children's language disorders, we would also want to know how dialects are learned by AAE- and SWE-speaking children with SLI relative to their same dialect–speaking TD peers. Consistent with studies of GAE and other languages, the SLI groups studied here overtly marked the tense and agreement structures in ways that reflected their respective dialects (AAE SLI: past 34%–46% > verbal –*s* 13%–30%; auxiliary BE past 59%–64% > auxiliary BE present 49%–57%; SWE SLI: past 34%–57%; verbal –*s* 26%–45%; auxiliary *is* 63%, *was* 59%, *were* 57% > *are* 43%). This linguistic information is important to understand so that dialect effects can be incorporated into assessment and treatment expectations. Indeed, in at least one treatment study of AAE-speaking children with SLI, dialect-specific language growth was evident (Smith & Bellon-Harn, 2015). Before treatment, the children, aged 4–5 years, produced 160 predicates that could support *is* or *are*, and their percentages of overt marking were 6% and 7%, respectively. Following treatment, their production of predicates increased to 384, and their percentages of overt marking were 47% and 21%, respectively, which showed not only gains in grammatical productivity but also higher percentages for *is* than *are*. In addition, the children's use of *is* with plural subjects (*they is…*) increased from 4% to 7%, which was another indication that the treatment, which was broad in nature, facilitated dialect-specific and dialect-appropriate language growth.

It is also informative to consider the five children with SLI who were excluded from the analysis of *was* and/or *were*. Of these children's 76 other responses on the auxiliary BE past probe (16 items × 5 children = 80 − 4 items coded as zero marked), 28 lacked a subject (e.g., *rock, rocking*), 15 included a subject but lacked progressive morphology on the verb (e.g., *she rock*), and 33 included the verb within a different grammatical context (e.g., *had rock, had rocking, is rocking, to rock*). None of these responses obligated *was* or *were,* so they could not be used to assess the children's grammatical productivity. However, the children's responses indicated that, although they understood the task (i.e., they knew they were to remember what they saw), they could not use the verbal prompts to generate the targeted predicate structure. Although these five children's ages, maternal education levels, and test profiles were like others in the SLI groups, on the other tense and agreement probes, they produced some of the highest percentages of other responses, and four of them produced some of the lowest percentages of overt marking. In other words, four of these children with SLI struggled with grammatical productivity, and all five struggled with the formulation of grammatically relevant predicate structure. Administration of the probes with strategic scoring allowed us to better understand these five children's linguistic weaknesses.

### Limitations and Future Directions

The study focused on children's overt marking of eight tense and agreement structures with one type of task and one type of strategic scoring approach. Additional studies are needed to examine other grammatical structures, tasks, and strategic scoring approaches. It may well be the case that very different scoring approaches will be needed for other grammatical structures and tasks. Recall that the current strategic scoring approach differed from the one implemented by Oetting et al. (2016). With the probes, we counted all Zero forms as unmarked and excluded responses coded as Other; with sentence recall, we counted three nonmainstream forms (i.e., zero verbal –*s*, *is* for *are*, and *was* for *were*) as correct and all others as incorrect. Scoring differences across studies were partly due to the nature of the tasks, but strategic scoring as a concept and approach is also very new and under development. Thus, we expect additional refinement of strategic scoring approaches as we test their clinical usefulness with other grammatical structures and tasks. Studies of different context manipulations may also lead to new insights on how best to create linguistic stimuli to maximize differences between clinical groups.

The study also was limited to kindergartners. Children's use of nonmainstream English forms changes with age (Horton-Ikard & Ellis Weismer, 2005; Van Hofwegen & Wolfram, 2010), and the tense and agreement deficit of children with SLI and other developmental language disorders also changes with age (Rice et al., 1998). Thus, future studies are needed to examine the tense and agreement systems of AAE- and SWE-speaking children who are younger and older than those studied here (as examples, see Hadley, & Short, 2005; Poll, Betz, & Miller, 2010). Finally, the study was limited to AAE and SWE as spoken in one rural southern community. Studies of children who speak AAE and SWE elsewhere and children who speak other dialects of English and/or are learning English as a second language are needed to examine the generalization of the findings (as examples, see Jacobson & Yu, 2018; Potapova, Kelly, Combiths, & Pruitt-Lord, 2018).

The current results also call for additional study of dialect-specific classification accuracy indices for existing tests and newly developed measures. As was shown here, different cut scores were identified as optimal for the probes when the dialects were separated, and overt marking of past tense (specifically past tense irregular) was identified as the best measure for differentiating the SLI and TD groups. Although an accuracy level of 76% (Se = .74, Sp = .77), which was observed for the past tense irregular items in AAE, is lower than desired, this accuracy level is higher than what Hendricks and Adlof (2017) found for AAE speakers on the CELF-5 with unmodified (−1 *SD* cut: Se = .88 and Sp = .48; −1.5 *SD* cut: Se = .76 and Sp = .67) and modified scoring (−1 *SD* cut: Se = .63 and Sp = .63; −1.5 *SD* cut: Se = .53 and Sp = .73). Recall also that we have classification accuracy information from three other tasks for the children studied here. Comparing across tasks and recalculating the classification accuracies using the same discriminant function analysis method as was done for the current probes, the strategically scored past tense irregular items yielded accuracy levels for the AAE-speaking children that were slightly better than what was found for two processing-based measures (size judgment AAE Se = .74 and Sp = .66; McDonald et al., 2018; nonword repetition, AAE Se = .54 and Sp = .77; McDonald & Oetting, 2019) but not as high as what was found for sentence recall (AAE Se = .77 and Sp = .86; Oetting et al., 2016).^5^ The sentence recall task also did not require different cut scores in AAE and SWE nor yield a dialect difference in classification accuracy. Given these findings, future studies should continue to use dialect-specific accuracy indices to evaluate and compare a wide range of tests and measures in isolation or when combined. Empirically derived cut scores, which may or may not need to be dialect-specific, should also be considered within these studies.

## Conclusions

The profile of SLI in AAE- and SWE-speaking kindergartners includes lower percentages of overt marking for tense and agreement than should be expected based on the performance of same dialect–speaking TD peers. To find clinical group differences on these structures, dialect-informed probes that are strategically scored are recommended. For the dialects combined and separated, the highest levels of classification accuracy with strategic scoring were found with the eight tense and agreement structures treated separately, the past tense probe, and the past tense irregular items. Given that dialect differences in classification accuracy also existed within the data and accuracy levels never exceeded 90%, the current tense and agreement probes should always be combined with other measures when making a diagnosis of SLI within the context of AAE or SWE. However, as descriptive measures, all four probes are recommended for AAE- or SWE-speaking children with SLI to help profile their linguistic strengths and weaknesses, determine treatment goals, and monitor treatment progress.

## Footnotes

1 The study also included 31 language samples from younger, language-matched TD controls, but these were not included within the discriminant function analyses that classified the children by their dialect and clinical status.

2 When sentences contained three functional categories, the SLI groups also made more errors involving negation than the TD groups, but these errors are not the focus here.

3 Accidental models from the examiner occurred 32 times, but only the 15 preceding an overtly marked form were excluded.

4 Although AAE and SWE allow nonmainstream overt verbal –*s* forms, these generally involve plural subjects (e.g., *They walks*) or serve as markers of historical past (e.g., *Yesterday he knocks on my door…*); the probe was not designed to elicit either of these verbal –*s* contexts.

5 In the original publications, classification accuracies for sentence recall, size judgment, and nonword repetition were based on calculations that maximized Youden’s J index, which yields slightly different results than a discriminant function analysis. To compare the results from these tasks to the current probes, classification accuracies were recalculated with discriminant function analyses. Although the results remained the same for size judgment, they changed slightly for the other two tasks (sentence recall: Dialect combined Se = .81, Sp = .87, AAE Se = .77, Sp = .86, SWE Se = .83, Sp = .89; nonword repetition: Dialects combined Se = .62, Sp = .85, AAE Se = .54, Sp = .77, SWE Se = .83, Sp = .94). Regardless of method, classification accuracies are highest for the sentence recall task.
